# Dual nature of human ACE2 glycosylation in binding to SARS-CoV-2 spike

**DOI:** 10.1073/pnas.2100425118

**Published:** 2021-04-26

**Authors:** Ahmad Reza Mehdipour, Gerhard Hummer

**Affiliations:** ^a^Department of Theoretical Biophysics, Max Planck Institute of Biophysics, 60438 Frankfurt am Main, Germany;; ^b^Institute for Biophysics, Goethe University Frankfurt, 60438 Frankfurt am Main, Germany

**Keywords:** molecular dynamics, ACE2 receptor, glycosylation, SARS-CoV-2, virus-host interaction

## Abstract

Binding of the spike protein of SARS-CoV-2 to the human angiotensin-converting enzyme 2 (ACE2) receptor triggers translocation of the virus into cells. Both the ACE2 receptor and the spike protein are heavily glycosylated, including at sites near their binding interface. We built fully glycosylated models of the ACE2 receptor bound to the receptor binding domain (RBD) of the SARS-CoV-2 spike protein. Using atomistic molecular dynamics (MD) simulations, we found that the glycosylation of the human ACE2 receptor contributes substantially to the binding of the virus. Interestingly, the glycans at two glycosylation sites, N90 and N322, have opposite effects on spike protein binding. The glycan at the N90 site partly covers the binding interface of the spike RBD. Therefore, this glycan can interfere with the binding of the spike protein and protect against docking of the virus to the cell. By contrast, the glycan at the N322 site interacts tightly with the RBD of the ACE2-bound spike protein and strengthens the complex. Remarkably, the N322 glycan binds to a conserved region of the spike protein identified previously as a cryptic epitope for a neutralizing antibody. By mapping the glycan binding sites, our MD simulations aid in the targeted development of neutralizing antibodies and SARS-CoV-2 fusion inhibitors.

## Significance

The SARS-CoV-2 virus infects cells by docking the spike protein at its surface to a receptor protein exposed on human cells. Both receptor and spike are covered by sugars. With molecular dynamics simulations, we show that sugars attached to the N90 site of the human receptor interfere with binding to the virus, explaining reports of increased susceptibility to infection if N90 glycosylation is lost. By contrast, sugars at the human receptor N322 site strengthen the binding to spike by latching onto a site on spike that is targeted also by neutralizing antibodies. By characterizing the contrasting roles of sugars in the interaction between virus and host cells, we aid in the targeted development of neutralizing antibodies and SARS-CoV-2 fusion inhibitors.

Angiotensin-converting enzyme 2 (ACE2) is an enzyme that catalyzes the hydrolysis of angiotensin II into angiotensin (1–7) to counterbalance the ACE receptor in blood pressure control (1). A single transmembrane helix anchors ACE2 into the plasma membrane of cells in the lungs, arteries, heart, kidney, and intestines (2). The vasodilatory effect of ACE2 has made it a promising target for drugs treating cardiovascular diseases (3).

ACE2 also serves as the entry point for several coronaviruses into cells, including SARS-CoV and SARS-CoV-2 (4–6). The binding of the spike protein of SARS-CoV and SARS-CoV-2 to the peptidase domain (PD) of ACE2 triggers endocytosis and translocation of both the virus and the ACE2 receptor into endosomes within cells (4). The human transmembrane serine protease 2, TMPRSS2, primes spike for efficient cell entry by cleaving its backbone at the boundary between the S1 and S2 subunits or within the S2 subunit (4). The structure of the ACE2 receptor in complex with the SARS-CoV-2 spike receptor binding domain (RBD) (7–9) reveals the major RBD interaction regions as helix H1 (Q24–Q42), a loop in a beta sheet (K353–R357), and the end of helix H2 (L79–Y83). With a 4-Å heavy-atom distance cutoff, 20 residues of ACE2 interact with 17 residues of the RBD, forming a buried interface of ∼1,700 Å^2^ (7).

The structure of full-length ACE2 has been resolved in complex with B^0^AT1 (also known as SLC6A19) (9). B^0^AT1 is a sodium-dependent neutral amino acid transporter (10). ACE2 functions as chaperone for B^0^AT1 and is responsible for its trafficking to the plasma membrane of kidney and intestine epithelial cells (11). Although it was speculated that B^0^AT1 prevents ACE2 cleavage by TMPRSS2 and thus could suppress SARS-CoV-2 infection (9, 12), other studies showed that SARS-CoV-2 can infect human small intestinal enterocytes where ACE2 is expected to be in complex with B^0^AT1 (13).

Both the ACE2 receptor and the spike protein are heavily glycosylated. Several glycosylation sites are near the binding interface (7, 9, 14, 15). Whereas the focus has largely been on amino acid interactions in the ACE2–spike binding interface (16, 17), the role of glycosylation in binding has been recognized (7, 18–20). The extracellular domain of the ACE2 receptor has seven N-glycosylation sites (N53, N90, N103, N322, N432, N546, and N690) and several O-glycosylation sites (e.g., T730) (9, 14). Among ACE2 glycosylation sites, the only well-characterized position regarding the effect on the spike binding and viral infectivity is N90. It is known from earlier SARS-CoV studies that glycosylation at the N90 position might interfere with virus binding and infectivity (21). Also, recent genetic and biochemical studies showed that mutations of N90, which remove the glycosylation site directly, or of T92, which remove the glycosylation site indirectly by eliminating the glycosylation motif (NXT), increase the susceptibility to SARS-CoV-2 infection (22, 23).

We use extensive molecular dynamics (MD) simulations to gain a detailed molecular-level understanding of how ACE2 glycosylation impacts the host–virus interactions. Glycosylation sites N90 and N322 of human ACE2 emerge as major determinants of its binding to SARS-CoV-2 spike. Remarkably, glycans at these sites have opposite effects, interfering with spike binding in one case, and strengthening binding in the other. Our findings provide direct guidance for the design of targeted antibodies and therapeutic inhibitors of viral entry.

## Results

### The RBD Is Stably Bound to ACE2.

**Fig. 1.**
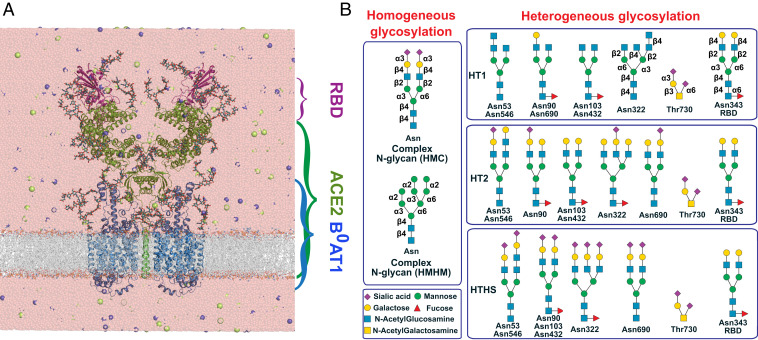
MD simulations of fully glycosylated B^0^AT1–ACE2–RBD complex. (*A*) System setup of the complex with explicit water and physiological concentration of ions. B^0^AT1, ACE2, and the RBD are shown in blue, green, and magenta cartoon, and glycans as licorice. (*B*) Distinct glycosylation patterns used in the simulations: two variants of homogeneous N-glycosylation (*Left*) and three variants of heterogeneous glycosylation (*Right*).

To gain molecular insight into the role of ACE2 glycosylation in spike–RBD binding, we performed MD simulations of a homodimeric membrane-anchored ACE2 receptor complexed with two spike RBDs and two B^0^AT1 (Fig. 1*A*). In addition, we simulated several variants of this complex: with and without ACE2 receptor, RBDs, and B^0^AT1 transporter (*SI Appendix*, Table S1). ACE2 has seven N-glycosylation sites (*SI Appendix*, Fig. S1) and one O-glycosylation site (13). B^0^AT1 carries five glycosylation sites, and the SARS-CoV-2 RBD one. For these glycosylation sites, we considered distinct glycosylation patterns: two variants of homogeneous N-glycosylation and three variants of heterogeneous glycosylation (Fig. 1*B* and *SI Appendix*, Table S1). For each of the 11 setups, we performed three independent MD simulation runs (1 × 1 μs and 2 × 480 ns). In each replicate, we built the glycans from scratch to minimize possible bias.

The ACE2–RBD complex was dynamic but remained stably bound in the simulations. The fraction of native interfacial amino acid contacts, as identified by cryo-electron microscopy (EM), was 80% on average and remained above 50% in all setups and trajectories (*SI Appendix*, Fig. S2*A*). In a visual inspection, no dissociation or initiation of dissociation was noticeable. The root-mean-square distance (RMSD) of the PD–RBD complex relative to the cryo-EM structure (Protein Data Bank [PDB] ID 6M17) was 3 Å on average and did not exceed 6 Å with the exception of one monomer of the HT1 system (*SI Appendix*, Fig. S2*B*). The low RMSD values of the complex (*SI Appendix*, Fig. S2*B*) imply that the PDs (residues 21–610) of the ACE2 receptor dimer and the RBDs were also internally stable. Of the two dimerization interfaces between the PDs in the ACE2 dimer, the lower interface with its extensive network of interactions kept on average 80% of the native contacts during the simulations, whereas the upper interface with only few interactions was floppy (*SI Appendix*, Fig. S3).

The bound RBDs kept the ACE2 complex near the conformation of the cryo-EM structure. With RBDs removed, the PDs approached each other (*SI Appendix*, Fig. S4). By contrast, the tilt of the PD relative to the TM domain is not sensitive to the glycosylation pattern or to binding of the RBD (*SI Appendix*, Fig. S4).

### B^0^AT1 Restricts ACE2 Conformational Dynamics and Covers Cleavage Site.

To gain insight on how B^0^AT1 might affect SARS-CoV-2 binding to ACE2, we studied the effect of B^0^AT1 on the structural dynamics and the accessibility of the binding site. We found that the presence of B^0^AT1 restricts the conformation space explored by the ACE2 receptor. In particular, the distribution of tilt angles with respect to the membrane normal is narrower with B^0^AT1 (*SI Appendix*, Fig. S4). We also found that bound B^0^AT1 restricts access to the ACE2 cleavage site (region 697–716) for serine proteases such as ADAM17 and TMPSSR2. The cleavage site is in close proximity to bound B^0^AT1. Using a 10-Å probe mimicking a protein domain like the head of TMPSSR2, we found an 80% reduction in accessibility of the cleavage site (*SI Appendix*, Fig. S5*A*). Both protein and glycans are involved in covering the cleavage site (*SI Appendix*, Fig. S5*B*). It has been shown that the TMPSSR2-mediated proteolysis of the ACE2 receptor enhances the coronavirus entry (4). Therefore, limiting the access of TMPSSR2 to the cleavage site of the ACE2 receptor may have a protective effect against viral infection. It remains to be seen where, beyond kidney and intestine epithelial cells (11), the complex between ACE2 and B^0^AT1 is maintained at the cell surface and how this interaction impacts viral entry.

### The N322 Glycan Strengthens RBD Binding.

Geometrically, the glycans at four glycosylation sites (N53, N90, N103, and N322) have the possibility to interact with the RBD. To quantify the interactions of each glycan with the RBD, we calculated the number of contacts between each glycan and the RBD and the associated interaction energy in the six setups where the RBD was present. Note that for each of these setups we have three independent simulations and each simulation has two copies of the RBD–ACE2 complex, giving us 3 × 2 = 6 copies to analyze for each interaction and a total of 6 × 6 = 36 copies to analyze for the RBD–ACE2 complex given the six different setups we simulated here. The total interaction energy between the ACE2 receptors and the RBD is between 890 and 1,040 kJ/mol, where two-thirds come from protein–protein interactions (580–680 kJ/mol) and one-third from ACE2–glycan interactions with the RBD (250–360 kJ/mol) (*SI Appendix*, Fig. S6).

**Fig. 2.**
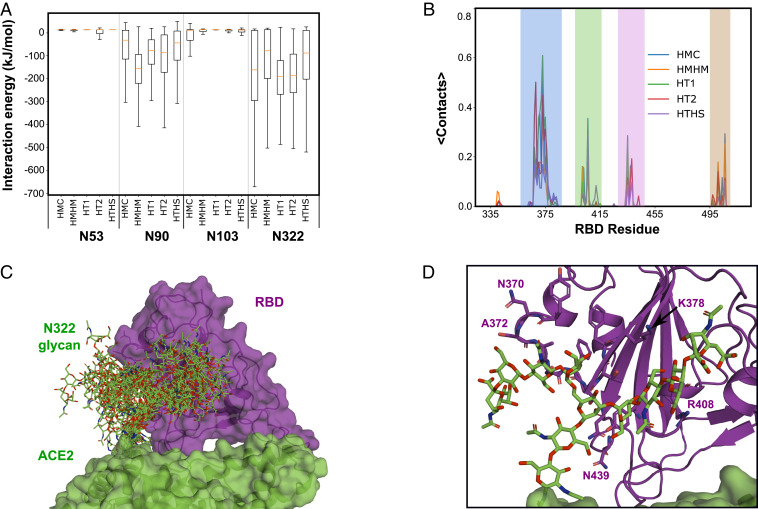
Interaction of ACE2 glycans with SARS-CoV-2 spike RBD. (*A*) Interaction energy between the ACE2 glycans and the RBD. The box for each glycan and MD setup shows the lower to upper quartile values of the data. The whiskers show the range of the data. The orange line is the median of the data. (*B*) Average number of residue–residue contacts between the N322 glycans and the RBD residues. Color shading highlights the four main interaction regions. (*C*) Simulation ensemble of the N322 glycan interacting with the RBD (from the HMC_WRBD_WBAT simulation). (*D*) Close-up of the interaction between the N322 glycan and the RBD in a representative snapshot of *C*.

The glycans at positions N90 and N322 interact most strongly with the RBD (Fig. 2*A* and *SI Appendix*, Fig. S7). The N322 glycan of ACE2 interacts tightly with the spike RBD in 24 out of the 36 RBD–ACE2 complexes simulated, forming 5 to 10 interactions on average (*SI Appendix*, Fig. S7*A*). In the remaining 12 complexes, interglycan interactions with the N546 glycan tend to drive the N322 glycan away from the RBD (*SI Appendix*, Fig. S8*A*). Consistent with the contact analysis, the median RBD–glycan interaction energy is favorable by 80 to 200 kJ/mol (Fig. 2*A*). The N322 glycan is also a major interaction partner of the N343^CoV2^ glycan on the RBD of the virus (*SI Appendix*, Fig. S8*B*). The N322 glycan on ACE2 and the N343^CoV2^ glycan on RBD have on average one to three interactions during the simulations. In 21 out of 36 RBD–ACE2 complexes, also the N90 glycan interacts tightly with the RBD, forming two to five interactions on average (*SI Appendix*, Fig. S7). The median interaction energy of 40–150 kJ/mol is less favorable than for the N322 glycan (Fig. 2*A*). By contrast, the glycans at sites N53 and N103 have on average less than one interaction in all complexes (Fig. 2*A*). However, the N53 glycan also has occasional, transient interactions with the N343^CoV2^ glycan.

In a comparison of the different glycosylation patterns, we found that the low-sialic (HT1, HT2) and high-mannose (HMHM) patterns show stronger interactions of the N90 and N322 glycan with the RBD, based on the number of interactions and the interaction energy. For the N322 glycan, the HT1 and HT2 glycans have the strongest interaction, whereas the high-mannose pattern produces the strongest interactions for the N90 glycan (Fig. 2*A* and *SI Appendix*, Fig. S7 *B* and *C*).

### The N322 Glycan Has a Specific Binding Site on the RBD.

Having identified the N322 glycan as strongly interacting with the RBD, we characterized its binding site on the RBD. The interaction map of glycan monosaccharides and RBD amino acids pinpointed regions in the RBD involving residues 365–387 and three smaller patches near residues 406–415, 435–445, and 499–508 (Fig. 2 *B* and *C*). The N322 glycan interacted mainly with Y369–K378, R408, N437, N439, and V503 near the site of the N501Y mutation associated with increased ACE2 binding affinity (24) and enhanced infectivity (Fig. 2*D*). Among these residues, the N322 glycan competes with the N90 glycan for the interaction with R408, a contact seen also in one of the experimental ACE2–RBD structures (8).

The N322 glycan binding site has a nonpolar core that is surrounded by several polar and charged residues (*SI Appendix*, Fig. S9). The interaction of the N322 glycan with the binding site is mainly governed by weak hydrogen bonds (C–H…N/O) with four to eight interactions per complex. Polar hydrogen bonds (two to four interactions per complex) and hydrophobic interactions (two to four interactions per complex) also contributed significantly to the binding affinity (*SI Appendix*, Table S2). The hydrophobic core of this binding site also explains its strong interactions with the less charged glycans of the high mannose (HMHM) and low sialic acid (HT1 and HT2) patterns (Fig. 2*A*).

### The N90 Glycan Shields ACE2 from RBD Binding.

**Fig. 3.**
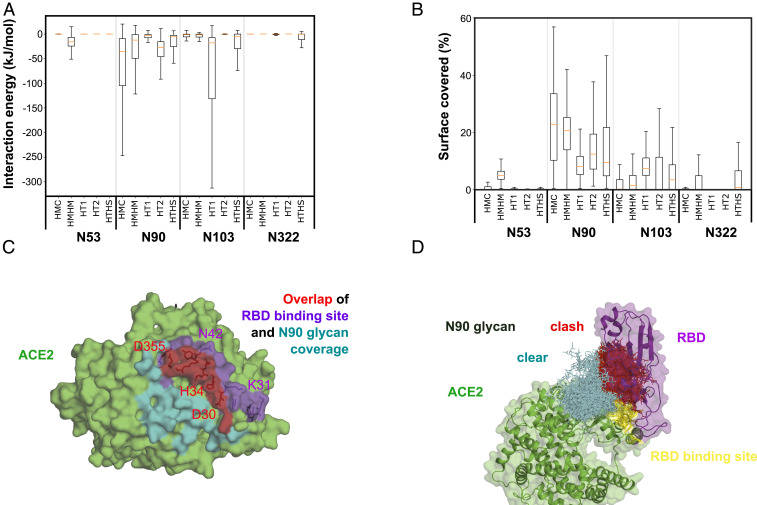
Interaction of ACE2 glycans with RBD binding site. (*A*) Interaction energy between the ACE2 glycans and the RBD binding site in the absence of the RBD. The box for each glycan and MD setup shows the lower to upper quartile values of the data. The whiskers show the range of the data. The orange line is the median of the data. (*B*) Fraction of RBD binding-site area covered by the N90 glycan from SASA calculations using a 5-Å probe for the HMC_WoRBD_WBAT setup. Results for 1.4- and 10-Å probe sizes are shown in *SI Appendix*, Fig. S11. (*C*) RBD binding site shielded by the N90 glycan. The RBD binding site, the area shielded by the N90 glycan, and their overlap are colored purple, cyan, and red, respectively. (*D*) Ensemble of the N90 glycan during the 1-µs-long simulation interacting with the RBD binding site in the HMC_WoRBD_WBAT setup. Steric clashes between glycan and RBD are illustrated by superimposing the RBD according to the HMC_WRBD_WBAT simulation. The RBD binding site is colored yellow. The glycans are shown in sticks. Glycans clashing with the RBD are colored red, and those without clashes are colored cyan.

The simulations of ACE2 without the RBD allowed us to explore the possibility that the glycans near the RBD binding site might block ACE2–RBD binding. First, we calculated the number of interactions between the glycan at each glycosylation site and the residues involved in RBD binding, as identified in the simulations of ACE2–RBD complexes. Note that for each of the five setups without RBD, we had three simulation replicates and in each simulation two copies of ACE2, giving us altogether 5 × 3 × 2 = 30 copies to analyze. Of all glycans on ACE2, the one at the N90 position interacted most strongly with the uncomplexed RBD binding site (Fig. 3*A*), forming one to two sugar–residue contacts typically (*SI Appendix*, Fig. S10). The other glycans had less than one contact on average. Interglycan interactions with the N322 glycan was the major factor keeping the N90 glycan away from the RBD binding site (*SI Appendix*, Fig. S10).

We further quantified the glycan coverage of the binding surface by comparing the solvent-accessible surface area (SASA) of the binding region in ACE2 in the presence and absence of each glycan. Using different probe sizes allowed us to investigate distinct types of blocking. Smaller probes (1.4 Å) detect the regions in the binding site that are in a direct contact with the glycan. Larger probes report on the accessibility of large molecules such as antibodies. The glycans covered on average 7.6% (2.3–16.7%), 25.9% (8.2–37.5%), and 41.5% (17.9–56.8%) of the binding site based on SASA calculations using probe sizes of 1.4, 5, and 10 Å, respectively (*SI Appendix*, Fig. S11). The N90 glycan was the main glycan blocking the binding site, covering 3.7% (0.4–10.9%), 16.2 (4.7–32.4%), and 26.8 (12.4–43.8%), respectively, for the three probe sizes (Fig. 3 *B* and *C*). As a result, an RBD superimposed onto ACE2 as seen in their complex would sterically clash with the N90 glycan in large fraction of configurations, especially near the binding interface (Fig. 3*D* and *SI Appendix*, Fig. S12). We conclude that the N90 glycan interferes with RBD binding by blocking the interface.

For the N90 glycan, the two homogenous glycosylation patterns (HMC and HMHM) showed higher coverage of the binding site (*SI Appendix*, Fig. S13). The main difference between homogenous and heterogenous glycosylation patterns at the N90 site is the presence of the fucose in the root of heterogenous patterns (Fig. 1*B*). The presence of fucose in this position appeared to restrict the flexibility of the whole glycan to rotate and to cover the binding site.

Compared to N90, the N322 and N103 glycans had a lower number of interactions with the binding site and covered a smaller fraction of its area (*SI Appendix*, Fig. S11). The N322 glycan in the high mannose (HMHM) or high sialic acid (HTHS) glycosylation patterns covered a patch of the binding site composed of residues E37, Y41, K353, and D355. The N103 glycan covered mainly Y82 and M83 and showed the highest coverage in the heterogenous glycosylation patterns (HT1, HT2, and HT3) (Fig. 3*B*).

### RBD–Glycan Interactions Are Spike-Conformation Sensitive.

Given their size, ACE2 glycans may interact also with parts of spike other than the RBD. To examine this possibility, we superimposed configurations along our MD trajectories into recently solved structures of the spike protein bound to the ACE2 receptor (PDB IDs 7A94, 7A95, 7A96, and 7A98) (25). The interactions of the glycans with spike clearly depend on spike conformation. For spike in the 3-up (PDB ID 7A98) or 2-up clockwise (PDB ID 7A95) conformations, which match the RBD in our complex with ACE2 most closely, we found ACE2 glycans to interact almost exclusively with the bound RBD. By contrast, in the 1-up (PDB ID 7A94) or 2-up anticlockwise conformations (PDB 7A96), both N90 and N322 glycan had direct interactions and clashes with the neighboring RBD domains while the NTD domain of the same subunit interacted with the ACE2 N322 and N564 glycans (*SI Appendix*, Fig. S14). A fully glycosylated model of the ACE–spike complex with spike in the 1-up conformation revealed possible interactions between the N17^CoV2^ glycan of spike and the ACE2 N546 glycan (and, less frequently, with the N322 glycan). The nearby N165^CoV2^ glycan of spike may interact with both ACE2 N322 and N546 glycans (*SI Appendix*, Fig. S15). Also, a recent study based on simulations of the ACE2–spike 1-up complex found that while the ACE2 N90 and N322 glycans interact with the spike, the N546 glycan interacts with the spike N74^CoV2^ and N165^CoV2^ glycans of the same subunit NTD (19). Therefore, a more detailed understanding of the role of the ACE2 glycans will require simulations of complexes between ACE2 and full-length spike in different conformations.

## Discussion

Viral and human proteins exposed at the outer surface of virions and cells, such as SARS-CoV-2 spike and human ACE2, are heavily glycosylated. However, on reconstituted proteins used for in vitro experiments, glycosylation typically lacks entirely or does not match the in situ pattern. Lacking detailed chemical and structural knowledge of the in situ glycan coat, the effect of glycosylation on complex formation is often ignored in modeling. MD simulations allow us to address this challenge and to explore the effects of a variable glycan coat on protein–protein interactions. Here we performed MD simulations of the fully glycosylated ACE2 receptor bound to the RBD of SARS-CoV-2 spike as well as unbound. These simulations gave us a detailed picture of the role of ACE2 glycans in the binding of the SARS-CoV-2 spike protein. The simulations showed contrasting effects of ACE2 glycosylation, weakening the binding of SARS-CoV-2 spike in case of the N90 glycan, strengthening the binding in case of the N322 glycan, and being neutral in case of other sites.

### N90 Glycosylation Protects against Infection.

The protective effect of the N90 glycan seen in our simulations is consistent with reports in the literature on infectivity dependence in human genetic variants. Studies after the SARS outbreak in 2003 showed that the N90 glycosylation might reduce infectivity (21). A recent deep mutational analysis demonstrated that any mutation in N90 directly or in its glycosylation motif (T92) increases the binding affinity for the SARS-CoV-2 spike protein (23). Based on an analysis of human ACE2 polymorphism, T92I is among the human ACE2 variants that were predicted to increase susceptibility (22). Species such as ferret, civet, and pig that lack the N90 site show efficient binding of their ACE2 to spike (*SI Appendix*, Fig. S1). Interestingly, if residues 90–93 from the civet sequence are introduced at the N90 glycosylation site of human ACE2, the SARS-CoV spike protein binds substantially more efficiently (21).

### N322 Glycan Locks into a Cryptic Binding Site Targeted by Neutralizing Antibodies.

In contrast to the N90 glycan, the N322 glycan aids RBD binding according to genetic analyses. In fact, almost all mutations removing this glycosylation site are detrimental to the binding of the RBD (23). Mouse and rat lack N322 glycosylation (*SI Appendix*, Fig. S1) and are among the species where SARS-CoV-2 and SARS-CoV do not bind via the ACE2 receptor (6, 21, 26). An exception is pangolin, where the ACE2 receptor can efficiently bind to the spike protein (26), yet the N322 position is mutated and cannot be glycosylated.

**Fig. 4.**
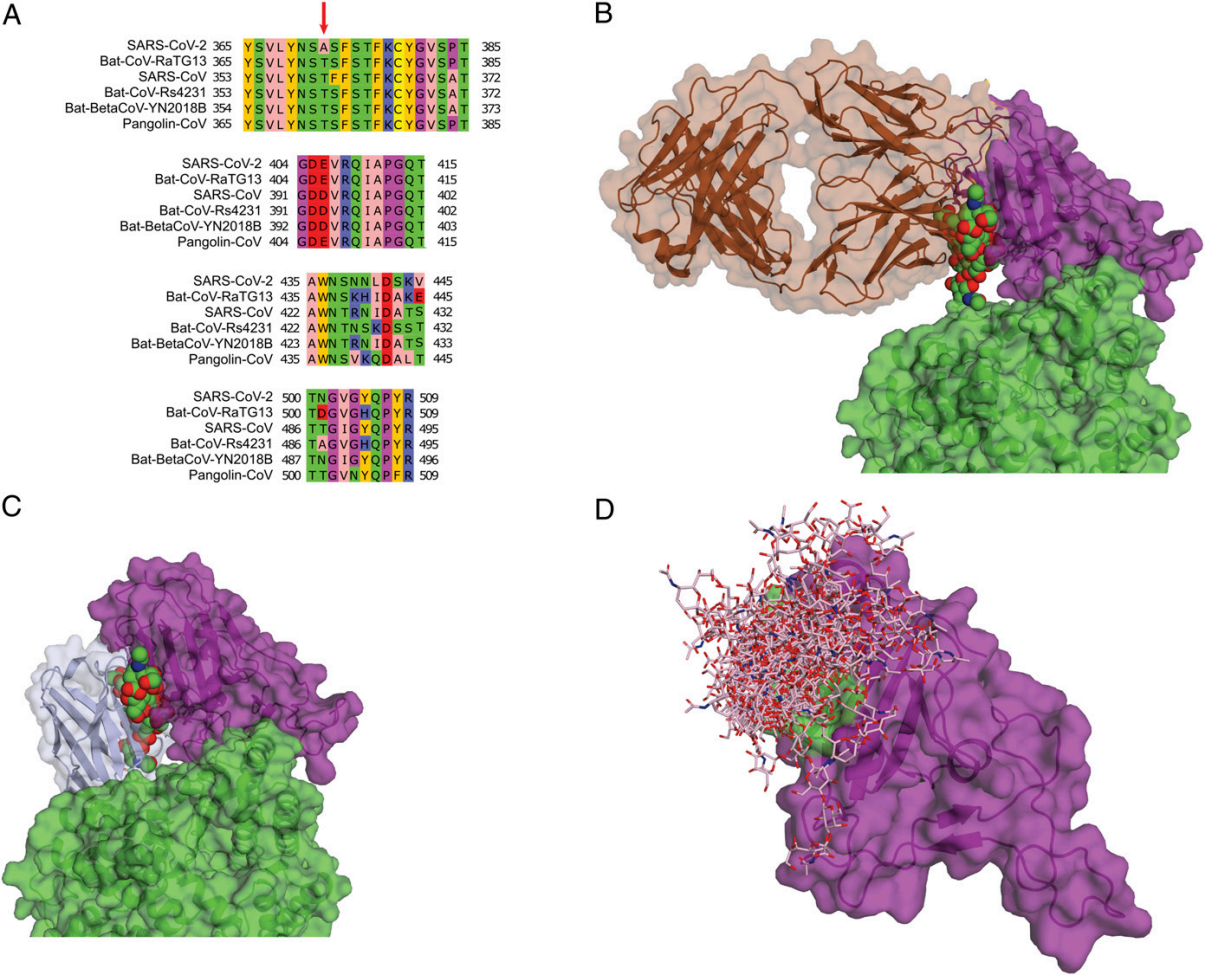
N322 binding site in RBD. (*A*) Sequence alignment of the N322 binding site in different coronaviruses. The red arrow indicates the difference between SARS-CoV and SARS-CoV-2 in the glycosylation motif of the N370^CoV2^ site. (*B*) Superimposition of antibody CR3022 (brown) targeting the cryptic epitope with a representative snapshot of the N322 glycan (space filling, green). (*C*) Superimposition of antibody VHH-72 (light blue) targeting the region near the cryptic epitope with a representative snapshot of the N322 glycan (space filling, green). (*D*) Ensemble of an artificially added N370^CoV2^ glycan (sticks) interacting with the N322 binding site (green) in the simulation of the RBD alone.

Our MD simulations show how the N322 glycan contributes to ACE2–spike binding and thus give us a mechanistic explanation for the genetic data. We found the N322 glycan to bind into a well-defined region of the RBD near residues 369–378. This binding site is mostly conserved among different coronaviruses (Fig. 4*A*). The site on the RBD binding the N322 glycan, as seen in our simulations, is a prime target for neutralizing antibodies. First, this region contributes positively to the binding of the spike protein, and second, in contrast to SARS-CoV, this region is not covered by a glycan and presumably exposed to the solvent (at least in some conformations of the spike protein).

The recent hunt for neutralizing antibodies against the SARS-CoV-2 spike protein revealed a presumably cryptic epitope in the RBD targeted by a class of antibodies. Remarkably, this cryptic epitope coincides with the binding site for the N322 glycan in our MD simulations. An antibody (CR3022), which has been obtained from a convalescent SARS-CoV–infected patient, has a binding site that overlaps significantly with that of the N322 glycan in our simulations (Fig. 4*B*) (27). Intriguingly, the lower affinity of RBD^CoV2^ for CR3022 is associated with the absence of the N370^CoV2^ glycan. The VHH-72 antibody developed for RBD^CoV^ has a similar binding site as CR3022 (Fig. 4*C*) (28). This antibody mainly interacts with Y356^CoV^ (Y369^CoV2^), S358^CoV^ (S371^CoV2^), K365^CoV^ (K378^CoV2^), C366^CoV^ (C379^CoV2^), R426^CoV^ (N439^CoV2^), and Y494^CoV^ (Y508^CoV2^), most of which interact with the N322 glycan in the RBD^CoV2^. Both antibodies have a suboptimal affinity for RBD^CoV2^, as they were raised against RBD^CoV^. This shows the need for developing neutralizing antibodies targeting specifically RBD^CoV2^. Therefore, details of the interactions between the N322 glycan and this epitope are central for a rational design of antibodies.

The spike conformation is another important factor for host glycan interactions of its RBD. Based on modeling of the interaction of the full-length spike with the ACE2 receptor, it is clear that the N322 glycan interaction with the cryptic epitope will mostly form in either the 3-up or the 2-up clockwise conformations of the spike, which is similar to what has been shown for CR3022 (27). This is further evidence for the idea that the N322 glycan and CR3022 compete for the same site. Different spike conformations likely account also for the fact that Zhao et al. (19) found the N322 glycan to interact with the spike protein outside the RBD whereas in our simulations the N322 glycan interacted with the RBD. In the simulations of Zhao et al., spike was in the 1-up conformation where the NTD domain of the same subunit blocks access for the N322 glycan to the RBD. By contrast, in our simulation setup resembling the 3-up conformation, the N322 glycan has full access to the RBD and the cryptic epitope.

### Lack of N370^CoV2^ Glycosylation Opens a Window onto Spike.

We also explored the effect of spike glycosylation on ACE2 binding. Indeed, antigen glycosylation governs host immune responses. In case of SARS-CoV-2, glycans cover almost the entire surface of the spike protein, suggesting that the virus can avoid the host immune system in a stealth fashion. In this sense, the absence of the N370^CoV2^ glycosylation site in the RBD of SARS-CoV-2 compared to SARS seems perplexing, as this mutation leaves a patch of the RBD exposed as a target for host immunological responses (Fig. 4*A*). In an MD simulation of RBD^CoV2^, we found an artificially added N370^CoV2^ glycan to interact with the same core of residues in the RBD (Y369–K378) as the N322 glycan (Fig. 4*D*).

In conclusion, we established a molecular picture for the role of ACE2 glycosylation in the binding of the SARS-CoV-2 that provides mechanistic insights. Our findings provide a basis for the rational development of neutralizing antibodies and small molecules that target the N322-glycan binding site in the RBD of the SARS-CoV-2 spike protein, and possibly of small molecules that mimic the protective effect of the N90-glycosylation variant on human ACE2.

## Materials and Methods

### ACE2–B0AT1–RBD Complex.

The coordinates of the ACE2–B0AT1–RBD complex were taken from PDB ID 6M17 (9) rerefined by Tristan Croll (https://drive.google.com/drive/folders/1bwJENqIgl8q2p_fVu_qTHPLsUlB927P0). The complex contains a dimer of the ACE2 receptor in complex with the RBD and also the B^0^AT1 transporter. The ACE2 receptor was simulated in the dimeric form and in the absence or presence of the RBD and also the B^0^AT1 transporter in different simulation setups (*SI Appendix*, Table S1).

### Glycosylation.

Based on the cryo-EM structure of the complex, the ACE2 receptor, the RBD, and the B^0^AT1 transporter contain seven (N53, N90, N103, N322, N432, N546, N690), one (N343), and five (N158, N182, N258, N354, N358) N-glycosylation sites, respectively. We considered five glycosylation patterns in the simulations (Fig. 1 and *SI Appendix*, Table S1). Two zinc ions in the peptidase binding sites of ACE2 were retained during the simulations. In two sets of systems, the glycosylation sites in the ACE2 receptor and the B^0^AT1 transporter were uniformly glycosylated with either a complex N-glycan or a high-mannose N-glycan (the homogenous glycosylation patterns in Fig. 1). In three sets of systems, each site in the ACE2 receptor was glycosylated with the most frequent N-glycan based on the mass spectrometry (MS) data (14) (the heterogeneous glycosylation patterns in Fig. 1). In the heterogeneous glycosylated systems, an O-glycosylation site (T730) was also glycosylated based on the MS data (14) (the heterogeneous glycosylation patterns in Fig. 1). The glycan at the glycosylation site in the RBD (N343^CoV2^) was added based on the MS analysis of the SARS-CoV-2 spike protein (15).

To minimize possible bias in the initial configuration of the glycans, each setup was simulated in triplicate with different initial configurations of the glycans. We set up the initial glycosylation using CHARMM scripts obtained from the Glycan Modeler module of the CHARMM-GUI webserver (29). For each glycosylation pattern, five representative glycan models were generated by CHARMM using average glycosidic torsion angles as obtained from the Glycan Fragment Database (GFDB) (30). A conformational sampling was conducted by rigid-body rotations of glycosidic angles. Trial conformations were accepted if they had less than five heavy-atom clashes (cutoff, 2.5 Å) to start and a lower glycan–protein interaction energy after a 10-step steepest descent energy minimization using the CHARMM force field. This sampling ensured proper stereochemistry and removed remaining clashes (31, 32). After the sampling, the glycosylation models were visually inspected for remaining issues.

### System Setup.

The interaction of the ACE2 receptor with the RBD of the spike protein was studied with all-atom explicit solvent MD simulation using GROMACS v2019.6 (33). The lipid bilayers of palmitoyl oleoyl phosphatidyl-choline (POPC), palmitoyl oleoyl phosphatidyl-ethanolamine (POPE), palmitoyl oleoyl phosphatidyl-serin (POPS), palmitoylsphingomyelin (PSM), and cholesterol lipids were created using the CHARMM-GUI webserver (34). Lipid ratios are listed in *SI Appendix*, Table S3. These systems were solvated with water and 150 mM NaCl, resulting in boxes of ∼21 × 21 × 26 nm^3^ (∼1,000,000 atoms).

After 2,000 steps of steepest descent energy minimization, the membrane patch was equilibrated first for 1 ns of MD simulation in an NVT ensemble with a 1-fs time step and then in an NPT ensemble (7.5 ns) with a 2-fs time step using a Berendsen thermostat and barostat (35). Three production simulations (one 1 µs and two 480 ns long) were run with a 2-fs time step at a temperature of 310 K and a pressure of 1 bar in an NPT ensemble using a Nosé-Hoover thermostat (36) and a semi-isotropic Parrinello-Rahman barostat (37) with a characteristic time of 5 ps and a compressibility of 4.5 × 10^−5^ bar^−1^.

The all-atom CHARMM36m force field was used for protein, lipids, and ions, and TIP3P was used for water molecules (38, 39). The MD trajectories were analyzed with Visual Molecular Dynamics (VMD) (40) and MDAnalysis package (41).

### RBD Glycosylation.

We performed MD simulations of an isolated RBD (chain F of PDB ID 6M17) glycosylated at positions N343^CoV2^ and N370^CoV2^. For this system, we used the homogenous complex glycan (Fig. 1 *B*, *Left*). The RBD was solvated with water and 150 mM NaCl, resulting in boxes of ∼12.5 × 12.5 × 12.5 nm^3^ (∼84,000 atoms). After 2,000 steps of steepest descent energy minimization, the system was equilibrated first for 0.5 ns of MD simulation in an NVT ensemble with a 1-fs time step and then in an NPT ensemble (8.5 ns) with a 2-fs time step using a Berendsen thermostat and barostat (35). A production run of 1 μs was run with a 2-fs time step at a temperature of 310 K and a pressure of 1 bar in an NPT ensemble using a Nosé-Hoover thermostat (36) and an isotropic Parrinello-Rahman barostat (37) with a characteristic time of 5 ps and a compressibility of 4.5 × 10^−5^ bar^−1^.

### Analysis.

#### Residue–sugar contacts.

A protein residue and a glycan sugar were considered to be in contact when at least one heavy-atom pair was within 3.5 Å in distance. Glycan monosaccharides were treated as individual sugars. For a hydrogen bond, two geometrical conditions had to be fulfilled: 1) The D–H...A distance had to be lower than 3.4 Å, and 2) the D–H...A angle had to be higher than 120°, with D and A the donor and acceptor atoms, respectively. For a hydrophobic interaction, two C or S atoms had to be within 4 Å of each other.

#### Fraction of native contacts.

Following Best et al. (42), two heavy atoms *i* and *j* are considered to form a native contact if their distance *r*_*ij*_^0^ in the cryo-EM structure is less than 4.5 Å. We then defined the fraction of native contacts *Q*(*X*) in a configuration *X* as follows:

$$Q\left(X\right)=\frac{1}{N}\sum_{\left(i,j\right)}\frac{1}{1+e^{\left[\beta \left(r_{ij}\left(X\right)-\lambda r_{ij}^{0}\right)\right]}},$$

where the sum runs over the *N* pairs of native contacts (*i*,*j*) and *r*_*ij*_(*X*) is the distance between *i* and *j* in configuration *X*. We set the smoothing and padding parameters to *β* = 5 Å^−1^ and *λ* = 1.8, respectively.

#### Interaction energy.

To quantify the strength of the interaction between the RBD and ACE2 glycans, the nonbonded interaction energy between the two species were computed using the GROMACS g_energy module. The short-range Coulombic and Lennard-Jones energies between the RBD and each ACE2 glycan were computed and the sum of these two terms gave the total interaction energy.

#### SASA.

The SASA was calculated for each atom. We extended its radius by the size of the probe and determined the area of the sphere exposed to solvent. Three different probe sizes of 1.4, 5, and 10 Å were used to measure the SASA. Using different probe sizes allows to investigate distinct types of blocking. Smaller probes (1.4 Å) detect the regions in the binding site that are in a direct contact with the glycan, whereas larger probes (5 and 10 Å) identify regions shielded by the glycan without direct interactions between the glycan and the protein and they are a better measure to check the accessibility of large molecules such as antibodies to the region. As an alternative, Sikora et al. (43) probed the accessibility by docking of antibody fragments.

#### Conformation dynamics of ACE2.

The distance between the center of mass of the PDs (residues 21–610) was calculated to measure the movement of the dimer PD relative to each other.

The tilting angle of the ACE2 receptor was measured as the angle between two vectors: The first vector is drawn between the centers of mass of the PD (residues 21–610) of the monomer and of the TM domain (residues 727–747), and the second vector is between the centers of mass of the whole PD of the dimer (residues 21–610 of both monomers) and of the TM domain (residues 727–747) (*SI Appendix*, Fig. S4).

### Sequence Alignment.

The sequences of the ACE2 receptor from various species and also the RBD of the different coronaviruses were aligned using T-COFFEE program (44).

## Supplementary Material

Supplementary File

## Data Availability

All study data are included in the article and/or supporting information.
