# Supplementary material for: Dual nature of human ACE2 glycosylation in binding to SARS-CoV-2 spike
Source: Proc Natl Acad Sci U S A. 2021 Apr 26;118(19):e2100425118. doi: 10.1073/pnas.2100425118 (PMC8126795; doi:10.1073/pnas.2100425118)
Supplement: Supplementary File [file pnas.2100425118.sapp.pdf]

PNAS
www.pnas.org

Supplementary Information for

Dual nature of human ACE2 glycosylation in binding to SARS-CoV-2 spike

Ahmad Reza Mehdipour[a,*] and Gerhard Hummer[a,b*]
[a] Department of Theoretical Biophysics, Max Planck Institute of Biophysics, Max-von-Laue Str. 3, 60438 Frankfurt am Main, Germany
[b] Institute for Biophysics, Goethe University Frankfurt, 60438 Frankfurt am Main, Germany

*To whom correspondence should be addressed.
(Email: Ahmadreza.Mehdipour@biophys.mpg.de and Gerhard.Hummer@biophys.mpg.de)

This PDF file includes:

Figures S1 to S15
Tables S1 to S3

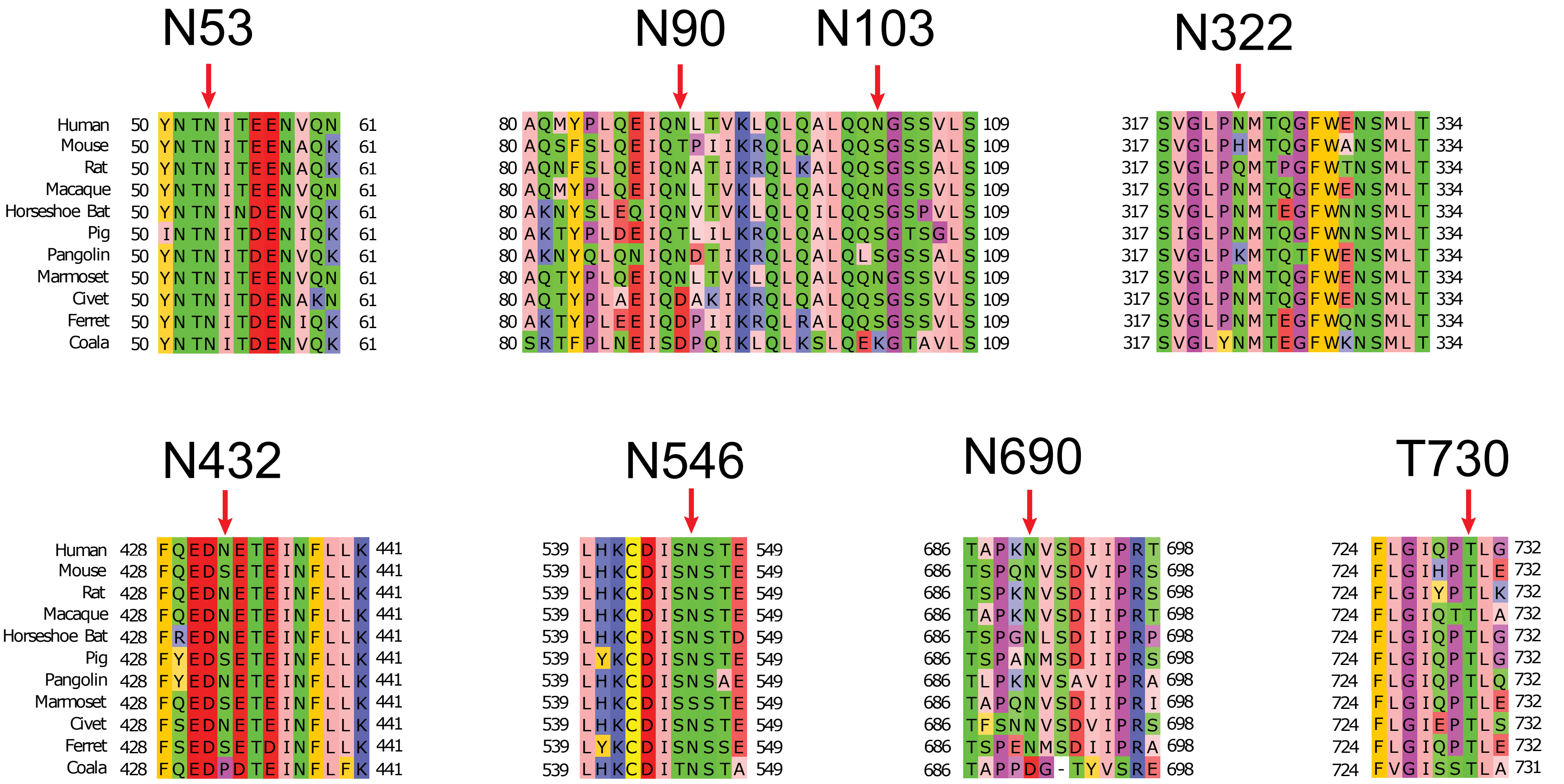

Figure S1. Amino acid sequence alignment of the ACE2 receptor (glycosylation sites) in human and selected species. The red arrows indicate the glycosylation sites in human ACE2 and the homologous positions in other species.

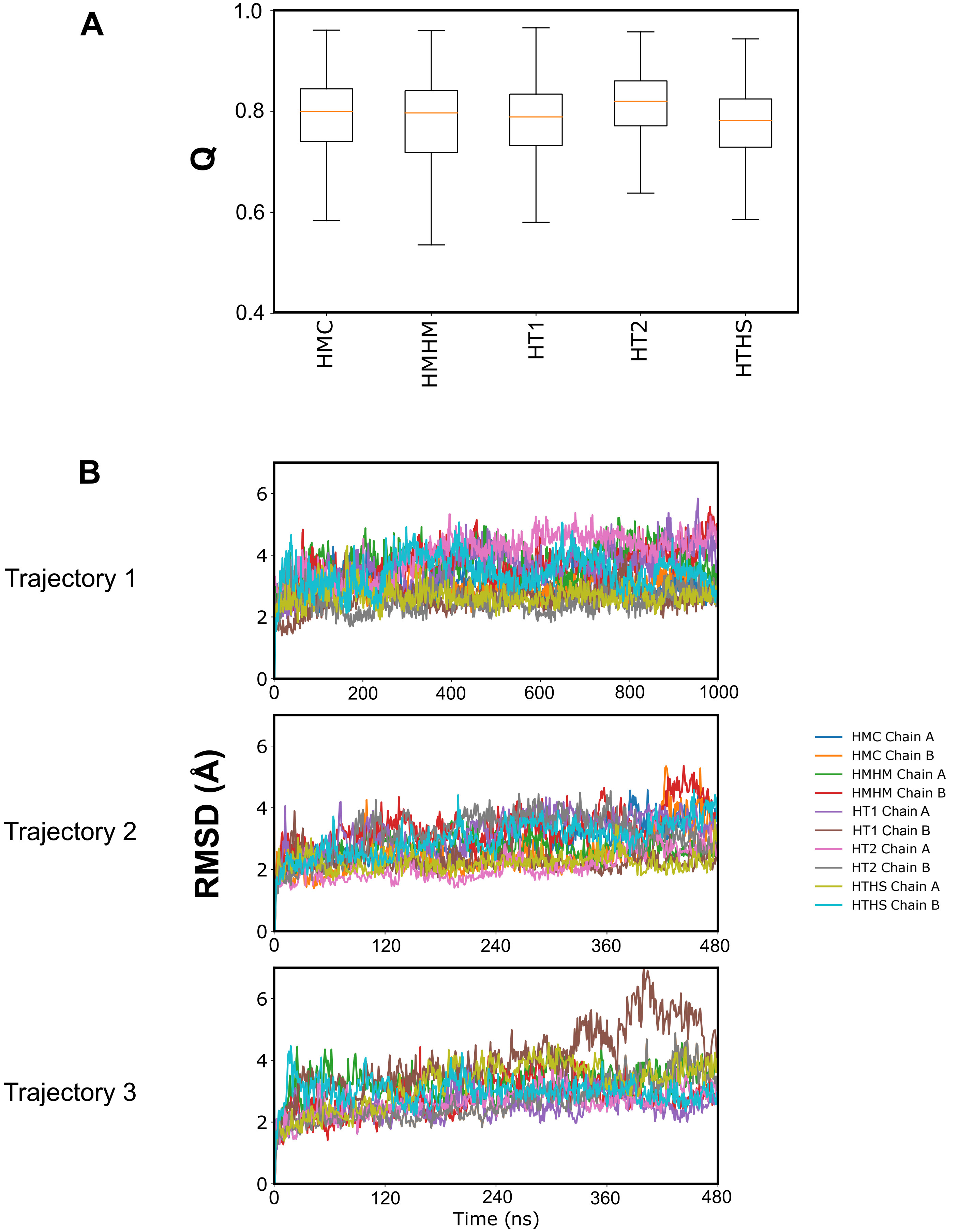

Figure S2. Structural and conformational stability of the ACE2 complex. A) The distribution of the fraction of native contacts (Q) for the binding interface between ACE2 and the RBD in different simulation setups. Boxes and whiskers indicate the interquartile range and the range of the data, respectively. The orange line is the median of the data. B) The time trace of the Cα RMSD of the ACE2 and RBD complex (the peptidase domain of ACE2 residues 21-710 and the whole RBD). The complex was first superimposed into the cryo-EM structure using the Cα atoms and then the RMSD was calculated.

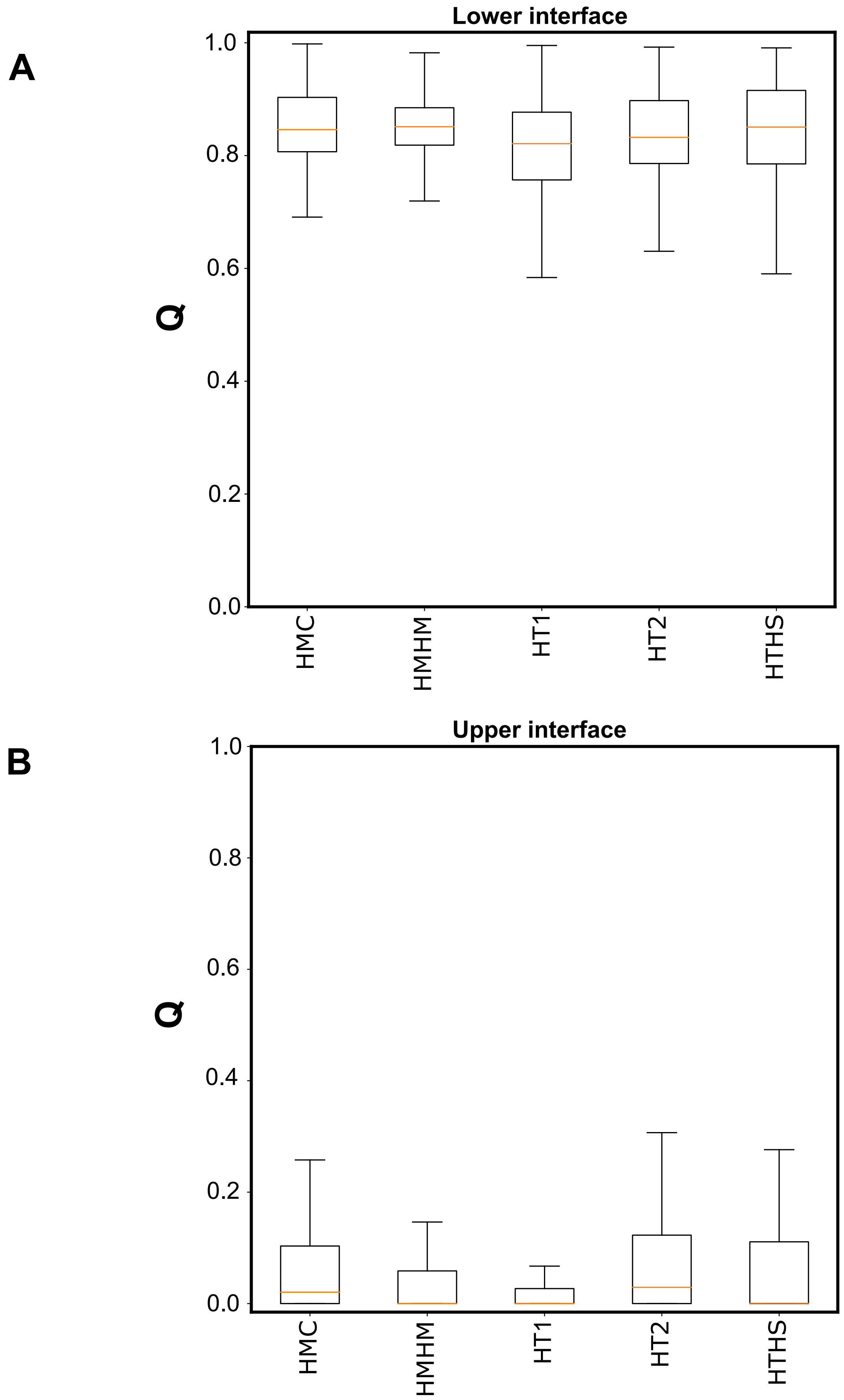

Figure S3. Conformational stability of the ACE2 dimerization interfaces. The distribution of the fraction of native contacts (Q) is shown between two ACE2 monomers at the lower (residues 610-725) and upper (residues 110-195) interfaces during in different simulation setups (A and B panels respectively). Boxes and whiskers indicate the interquartile range and the range of the data, respectively. The orange line is the median of the data.

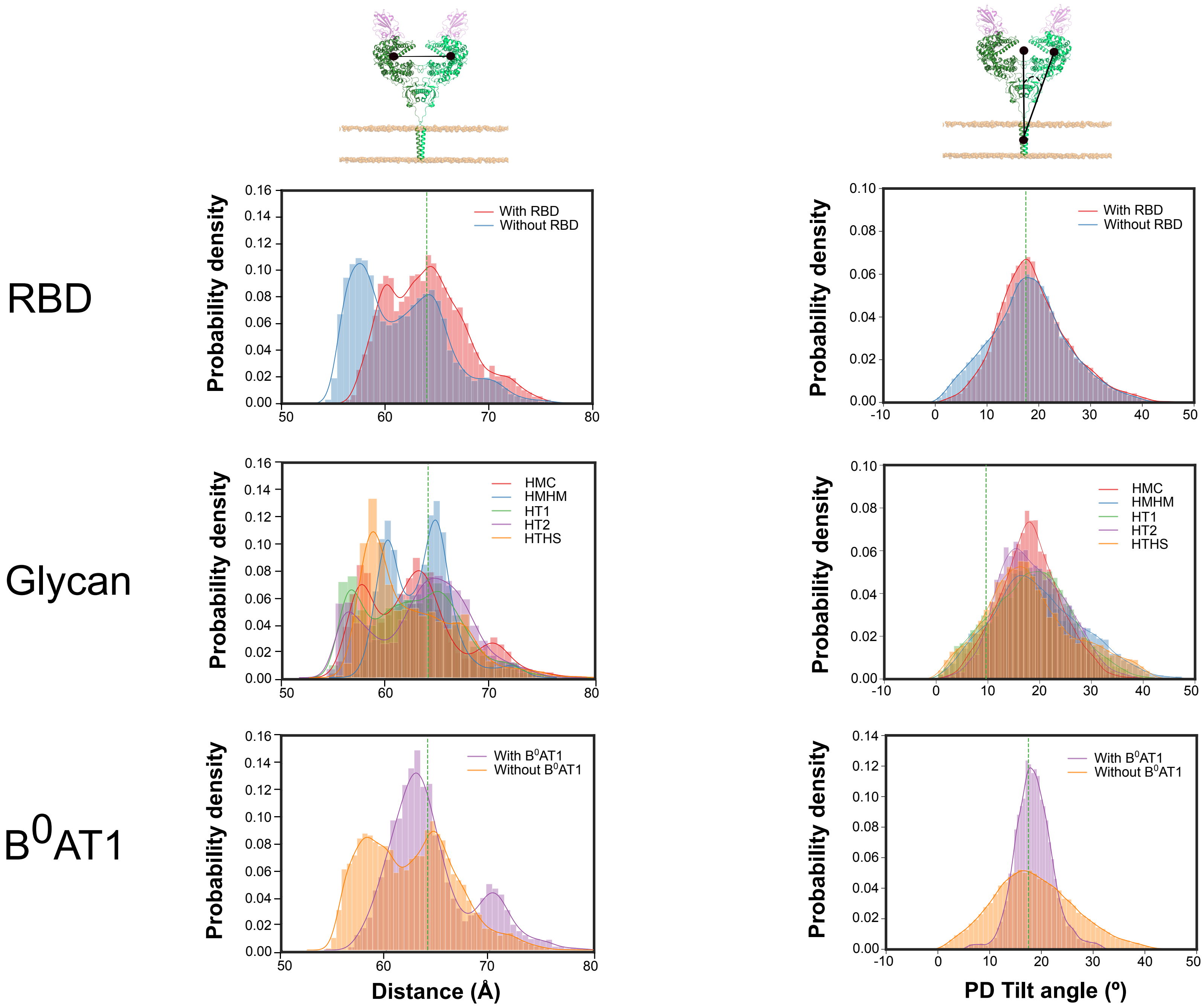

Figure S4. Conformational dynamics of ACE2 complexes with spike RBD and B^0AT1. Left column panels show the distance between the centers of mass of the peptidase domains (residues 21-610) in different simulation setups. Right column panels show the tilting angle of the ACE2 receptor with respect to the membrane normal. The green line shows the values for the cryo-EM structure. The lines are the representations of a kernel-density estimate of the probability density function (PDF) of each data set.

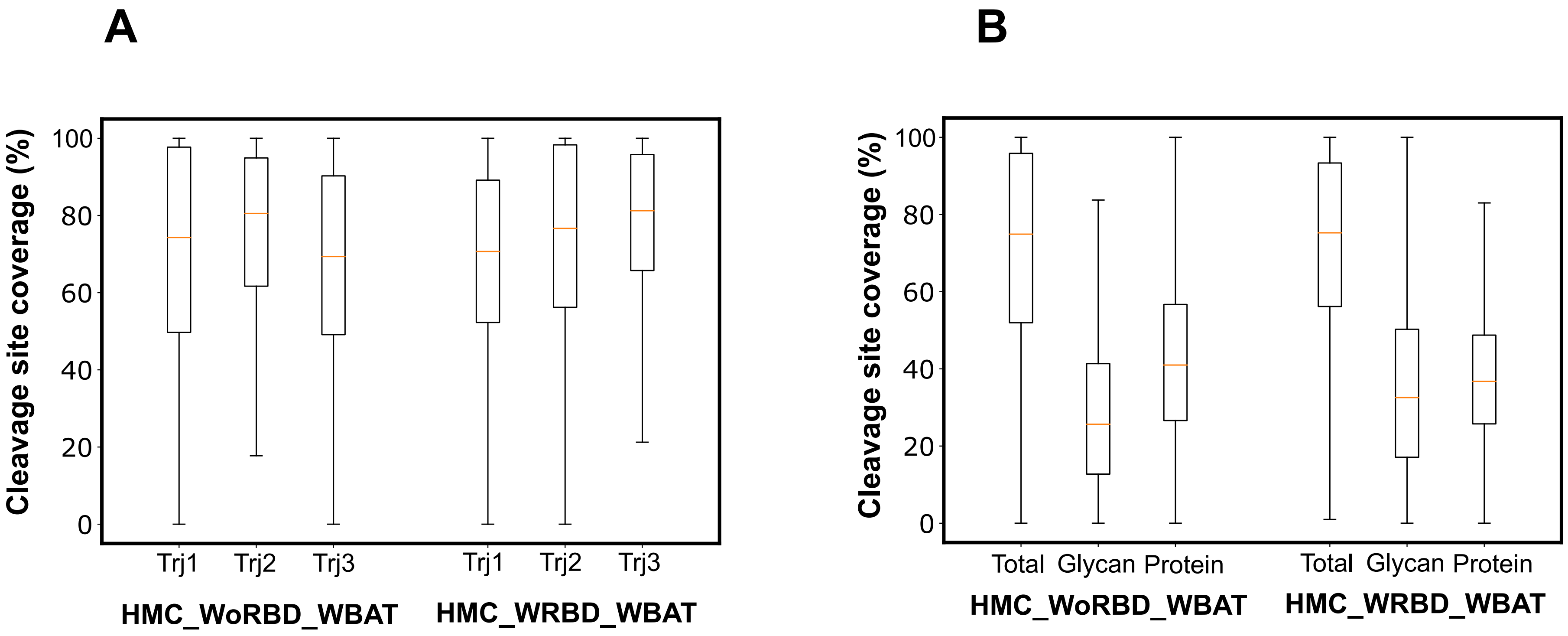

Figure S5. Protection of the ACE2 cleavage site by B^0AT1. A) shows the coverage of the cleavage site by B^0AT1 in different simulations runs. B) shows the contribution of protein and glycan in covering the cleavage site. Boxes and whiskers indicate the interquartile range and the range of the data, respectively. The orange line is the median of the data.

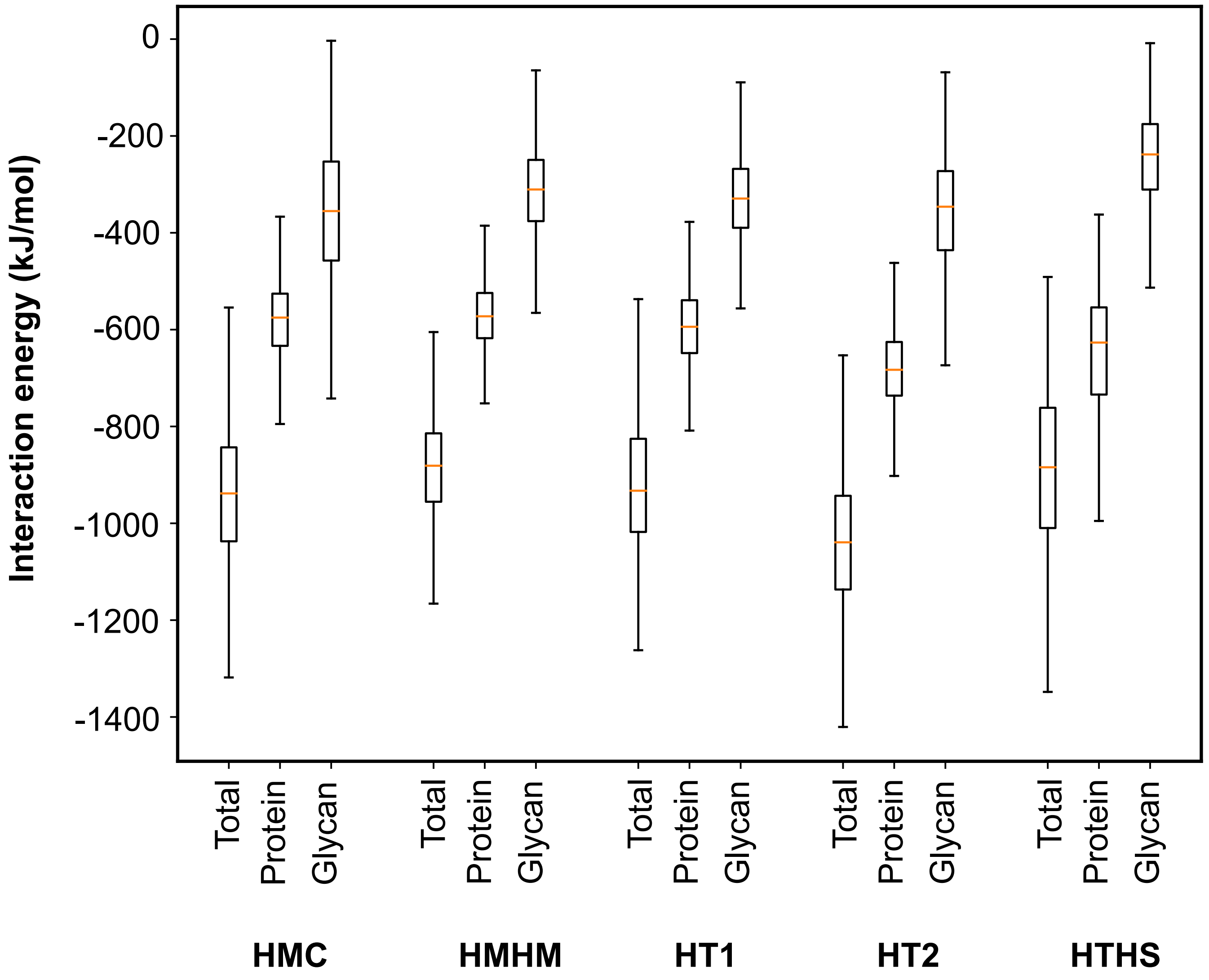

Figure S6. Interaction energy between ACE2 and RBD and the contribution of protein and glycans for each glycosylation pattern. Boxes and whiskers indicate the interquartile range and the range of the data, respectively. The orange line is the median of the data.

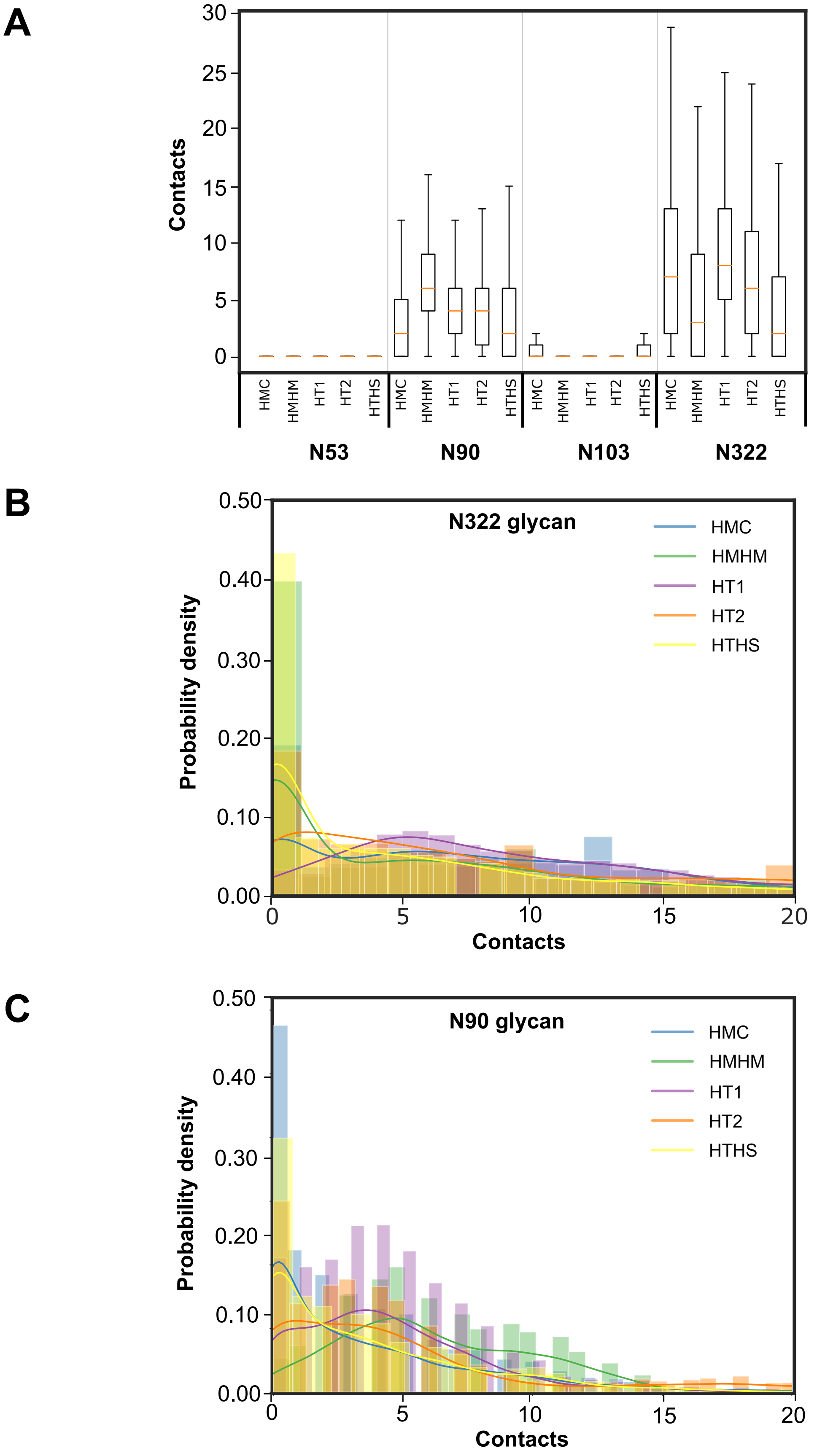

Figure S7. Interaction between the ACE2 glycans and the RBD. A) Number of residue-residue contacts is shown for four glycosylation sites near the RBD binding site for different glycosylation patterns. The other three glycosylation sites had no contact with the RBD. Boxes and whiskers indicate the interquartile range and the range of the data, respectively. The orange line is the median of the data. B) The distribution of number of residue-residue contacts for the N322 glycan for different glycosylation patterns. C) The distribution of number of residue-residue contacts for the N90 glycan for different glycosylation patterns. The lines in Panels B and C are the representations of a kernel-density estimate of the probability density function (PDF) of each data set.

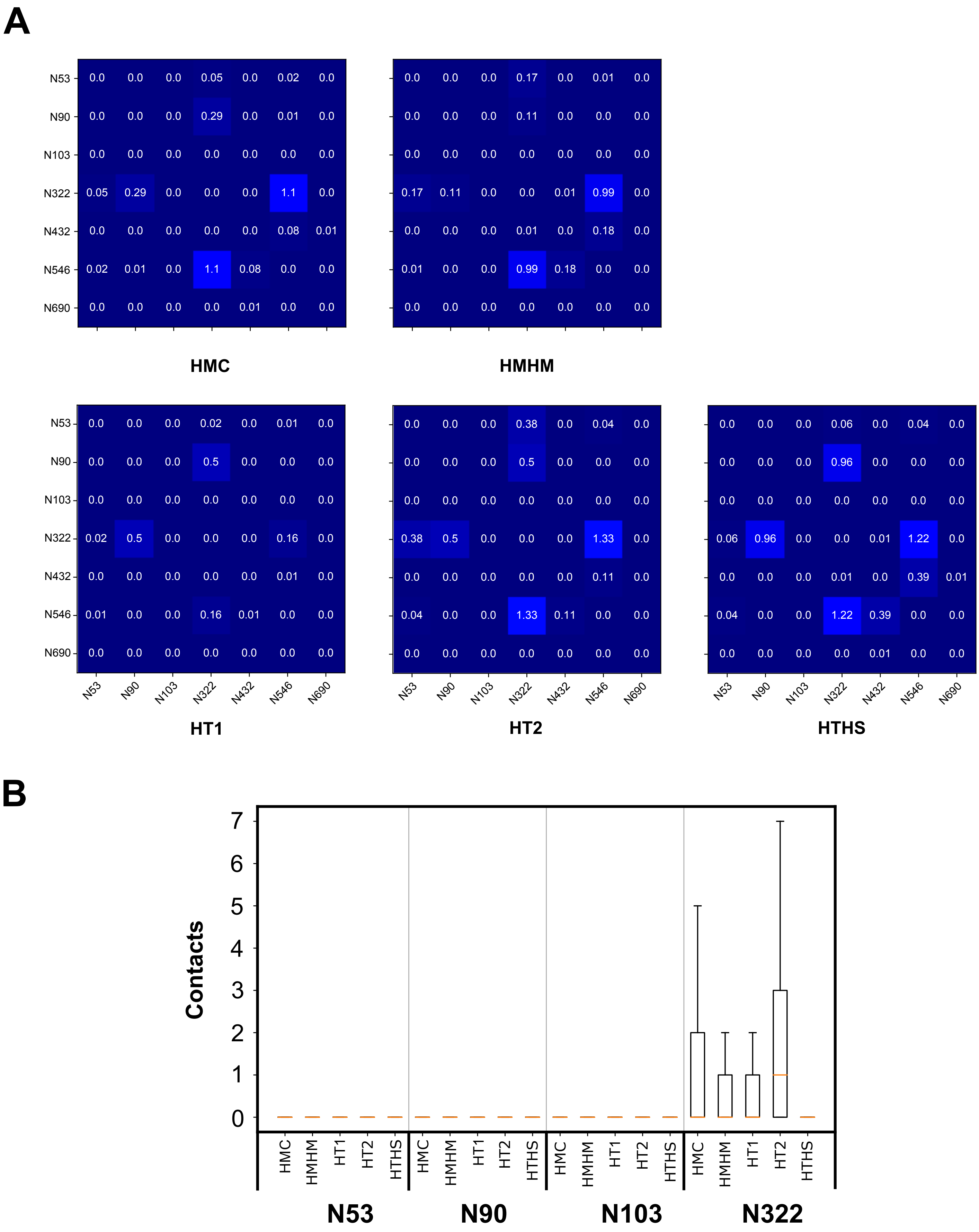

Figure S8. Inter-glycan interaction in ACE2-RBD complex. A) Average number of sugar-sugar contacts is shown for all glycan pairs in ACE2 for different glycosylation patterns (See Table S2) for those simulations performed in the presence of the RBD. B) Interaction between the ACE2 glycans and the N343^{CoV2} glycan. The number of sugar-sugar contacts is shown between 4 glycosylation sites in ACE2 near the RBD and the N343^{CoV2} glycan for different glycosylation patterns. Boxes and whiskers indicate the interquartile range and the range of the data, respectively. The orange line is the median of the data.

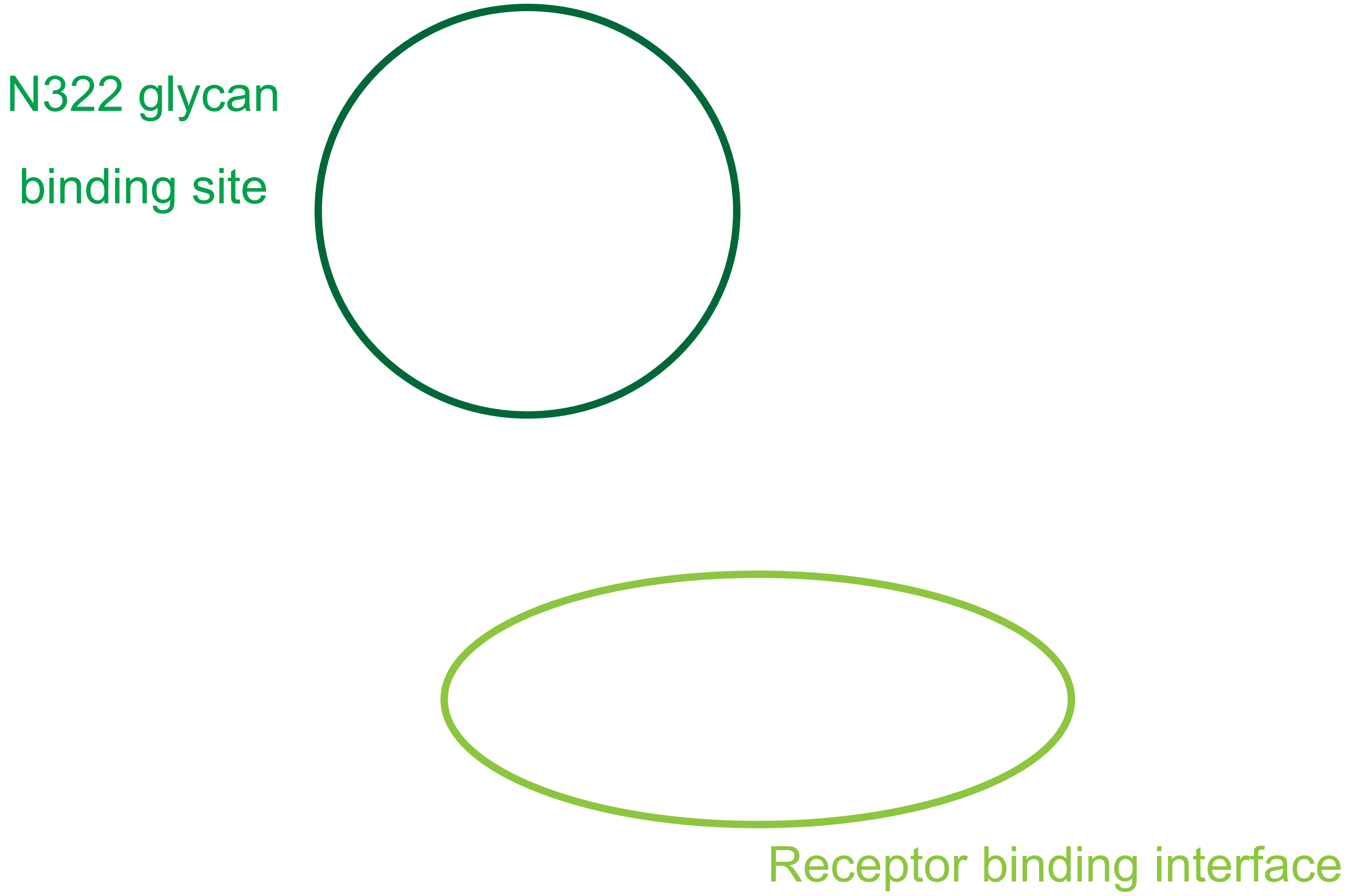

Figure S9. Electrostatic potential mapped onto the surface of the RBD. The binding site of the N322 glycan and interaction interface of ACE2 are marked by dark green and light green, respectively.

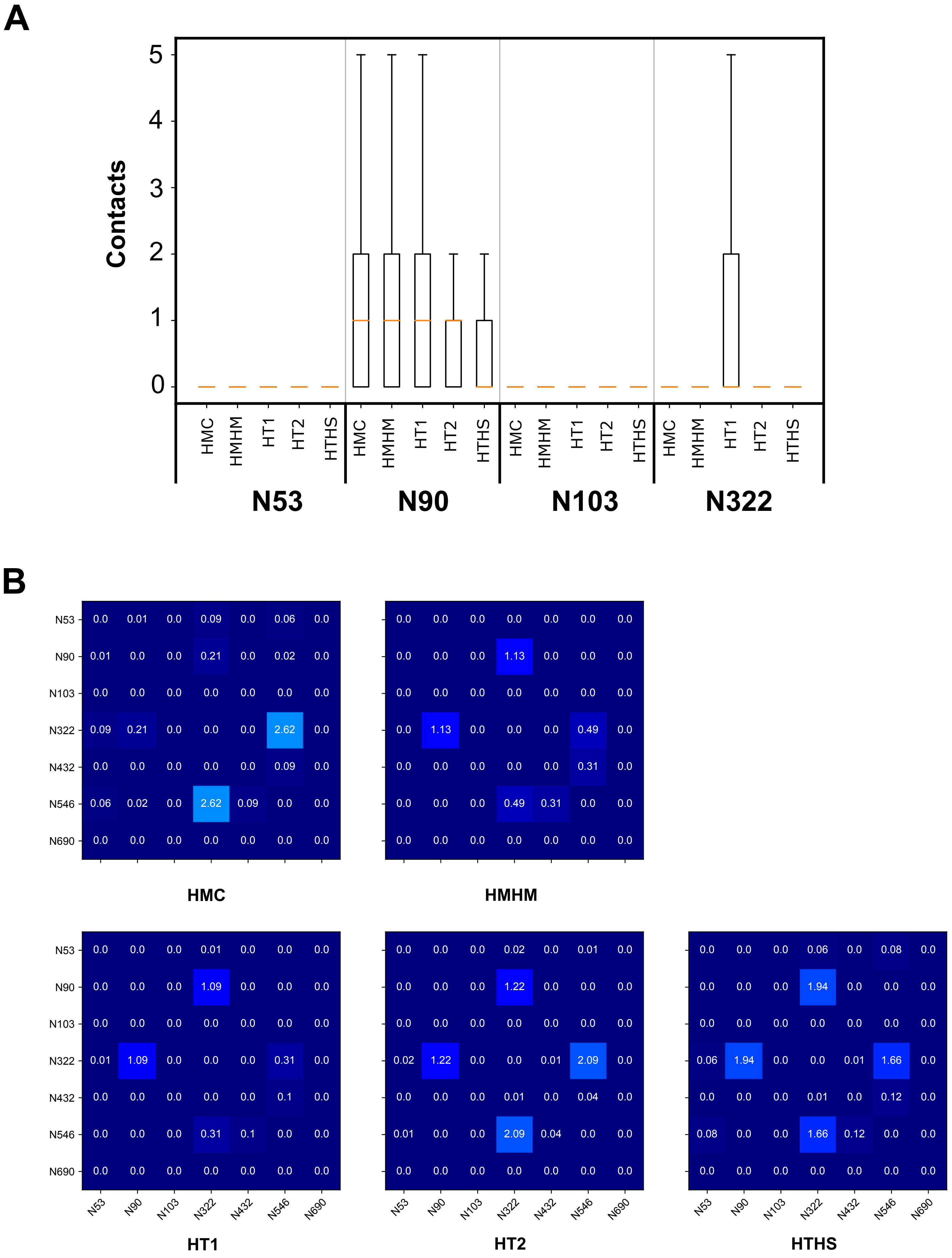

Figure S10. N90 glycan interaction in ACE2 complex. A) The number of sugar-residue contacts is shown for the N53, N90, N103, and N322 glycans in ACE2 with the RBD binding site for different glycosylation patterns (see Table S2) in simulations performed without bound RBD. Boxes and whiskers indicate the interquartile range and the range of the data, respectively. The orange line is the median of the data. B) The average number of sugar-sugar contacts is shown for all glycan pairs in ACE2 for different glycosylation patterns (See Table S2) for those simulations performed in the absence of the RBD.

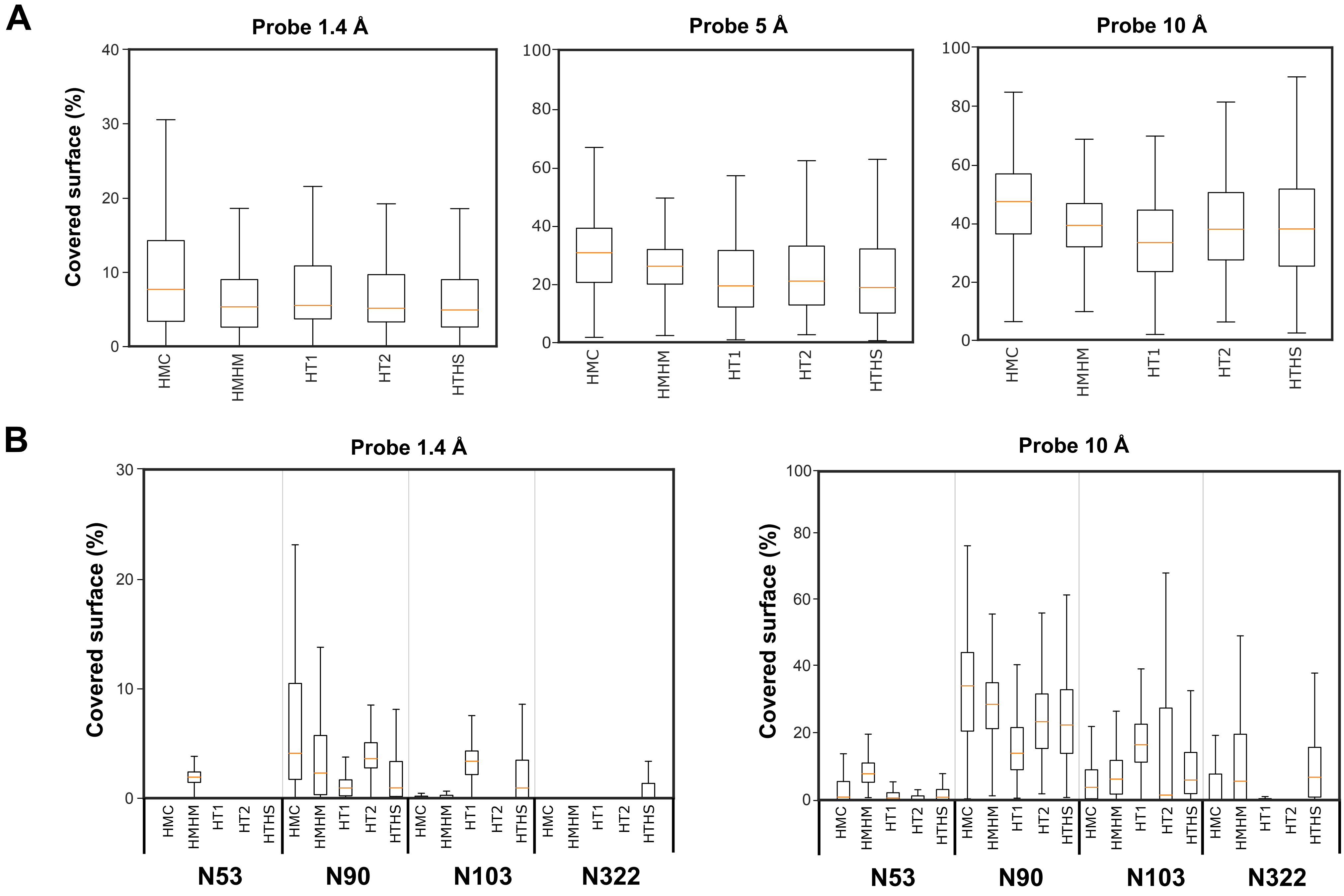

Figure S11. Coverage of the RBD binding site by ACE2 glycans. A) The coverage of the RBD binding site by ACE2 glycans in different simulation runs measured by three probes (1.4, 5, and 10 Å). B) The contribution of each glycan in covering the RBD binding site measured by two probes (1.4 and 10 Å). Boxes and whiskers indicate the interquartile range and the range of the data, respectively. The orange line is the median of the data.

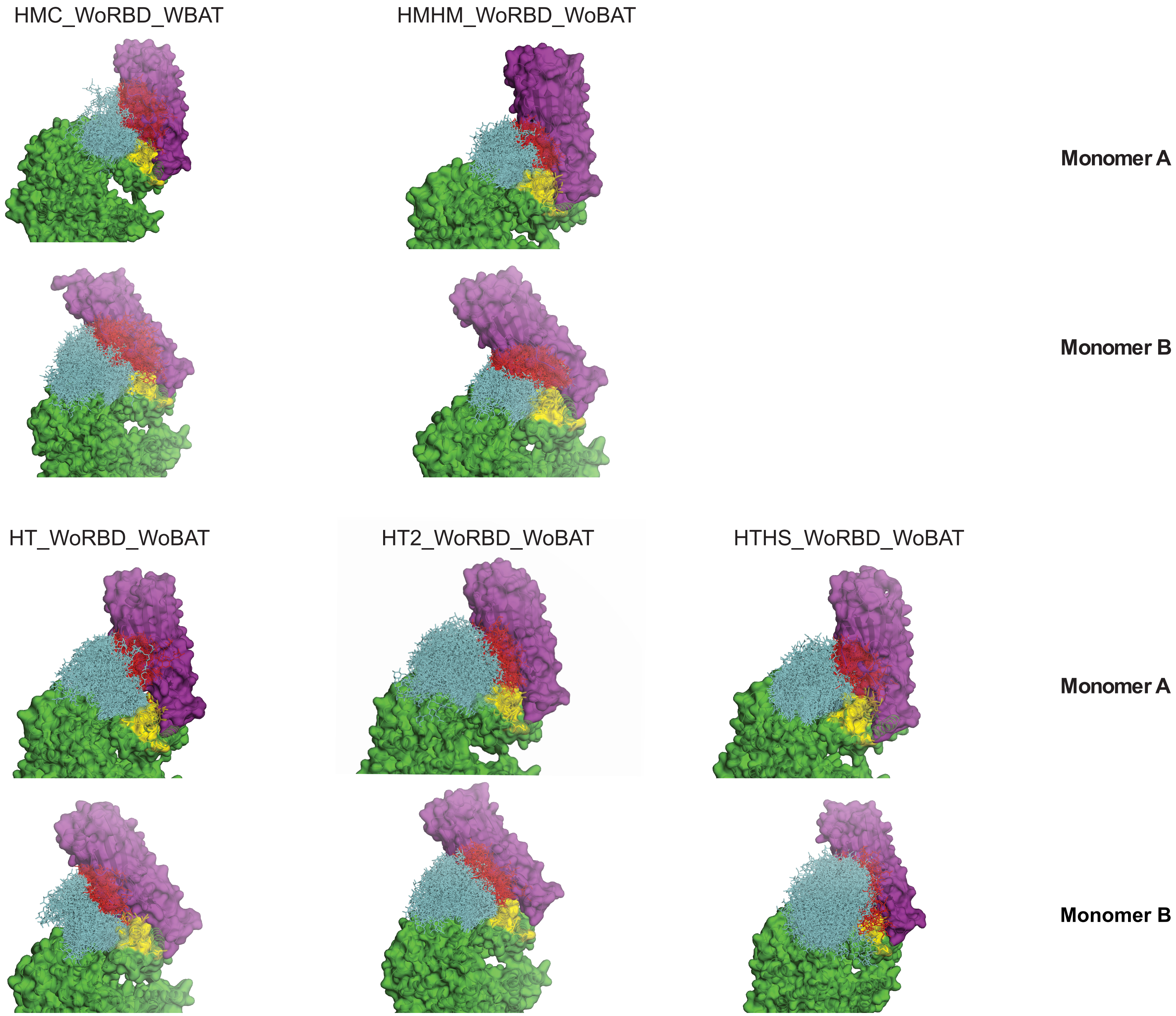

Figure S12. Interaction of N90 glycan with the RBD binding site during the simulations in the apo state of ACE2. The superimpose structure of the RBD from the corresponding RBD–bound simulation is also shown in each panel. The RBD binding site is colored yellow. The glycans are shown as sticks. Glycans that clash with the superimposed RBD are colored red and those without clashes are colored cyan.

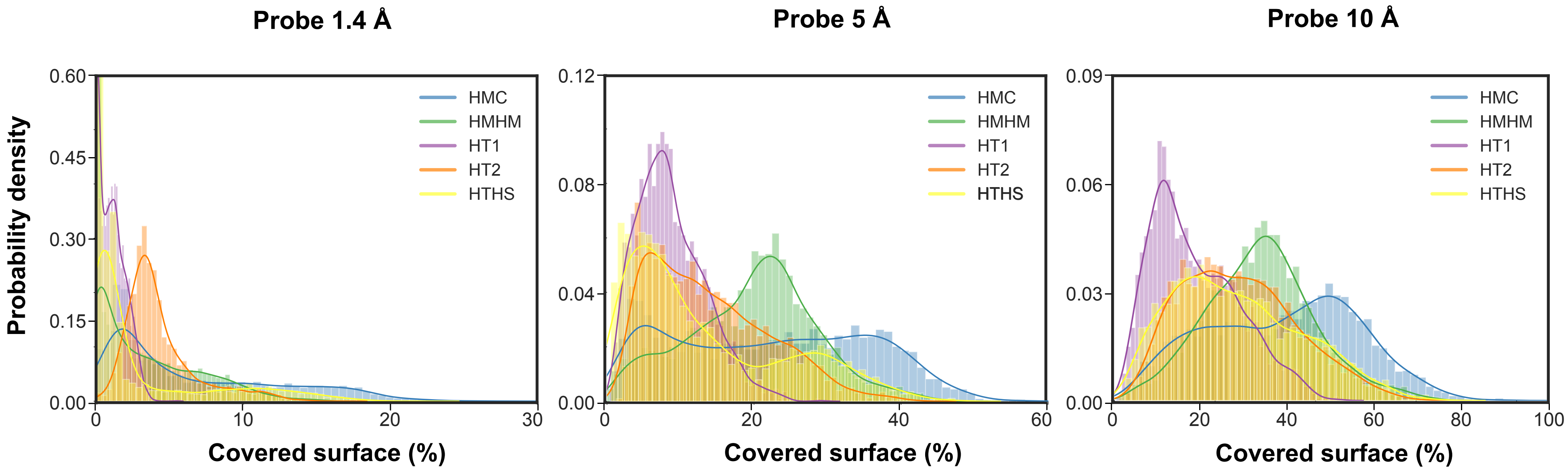

Figure S13. Effect of glycosylation pattern on the coverage of the RBD binding site by the N90 glycan is measured by probes 1.4, 5, and 10 Å. The lines are the representations of a kernel-density estimate of the probability density function (PDF) of each data set.

Spike in 1-up conformation

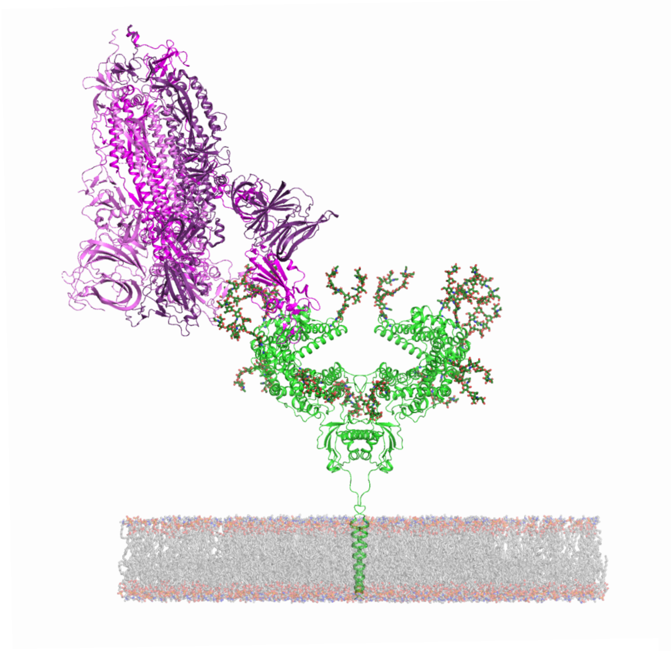

Spike in 2-up clockwise conformation

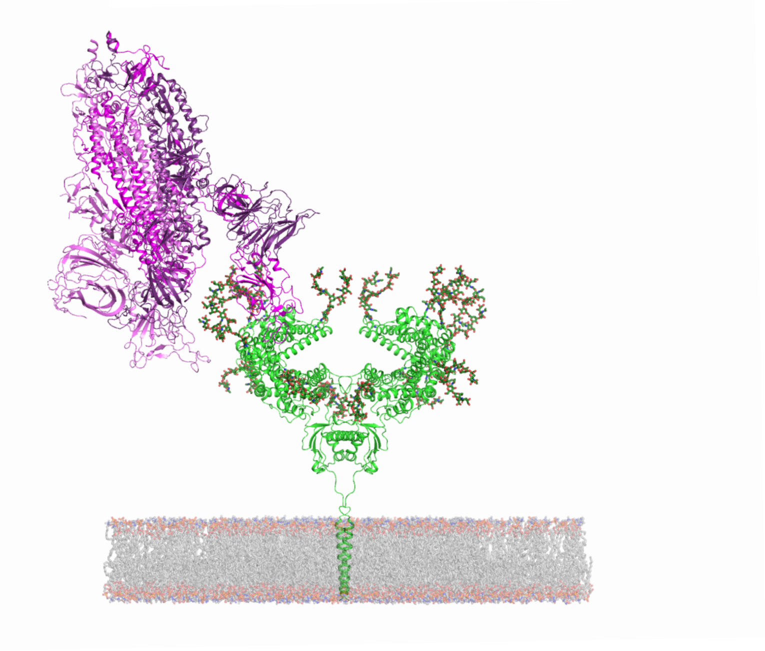

Spike in 2-up anti-clockwise conformation

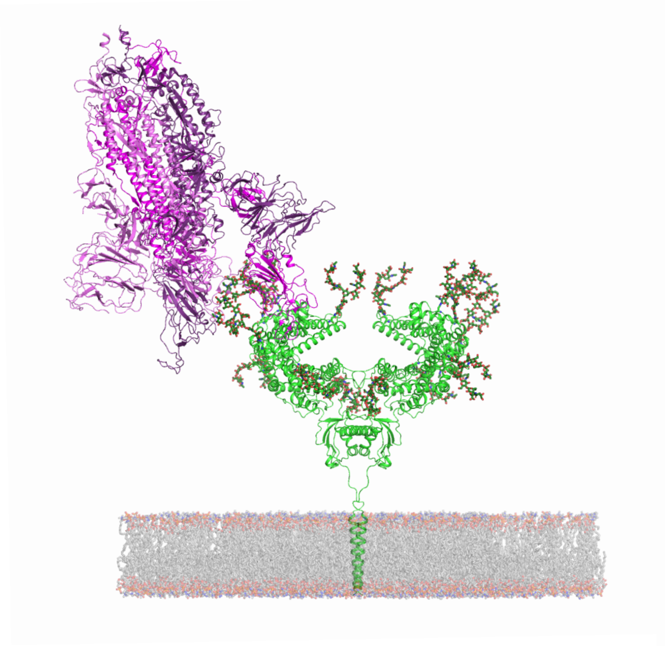

Spike in 3-up conformation

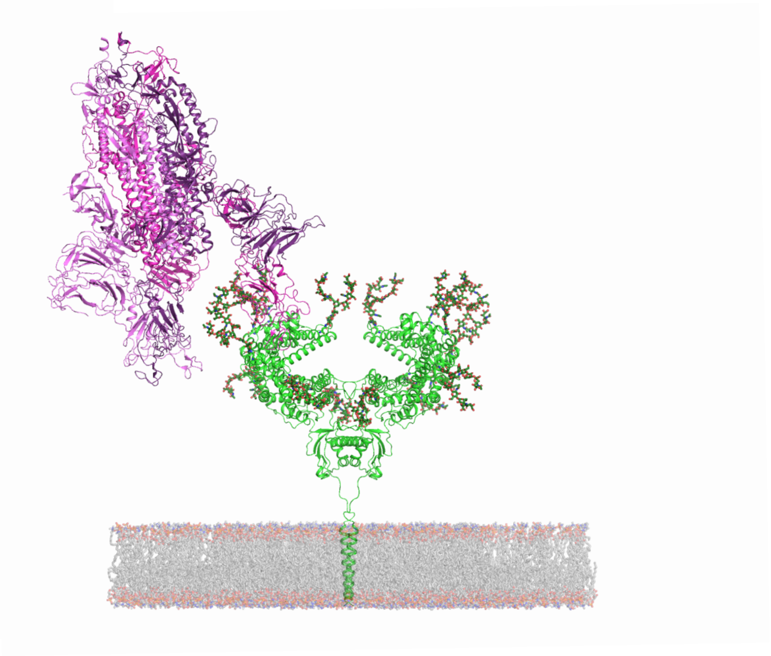

Figure S14. Effect of RBD conformation in spike on the interaction with ACE2 glycans.

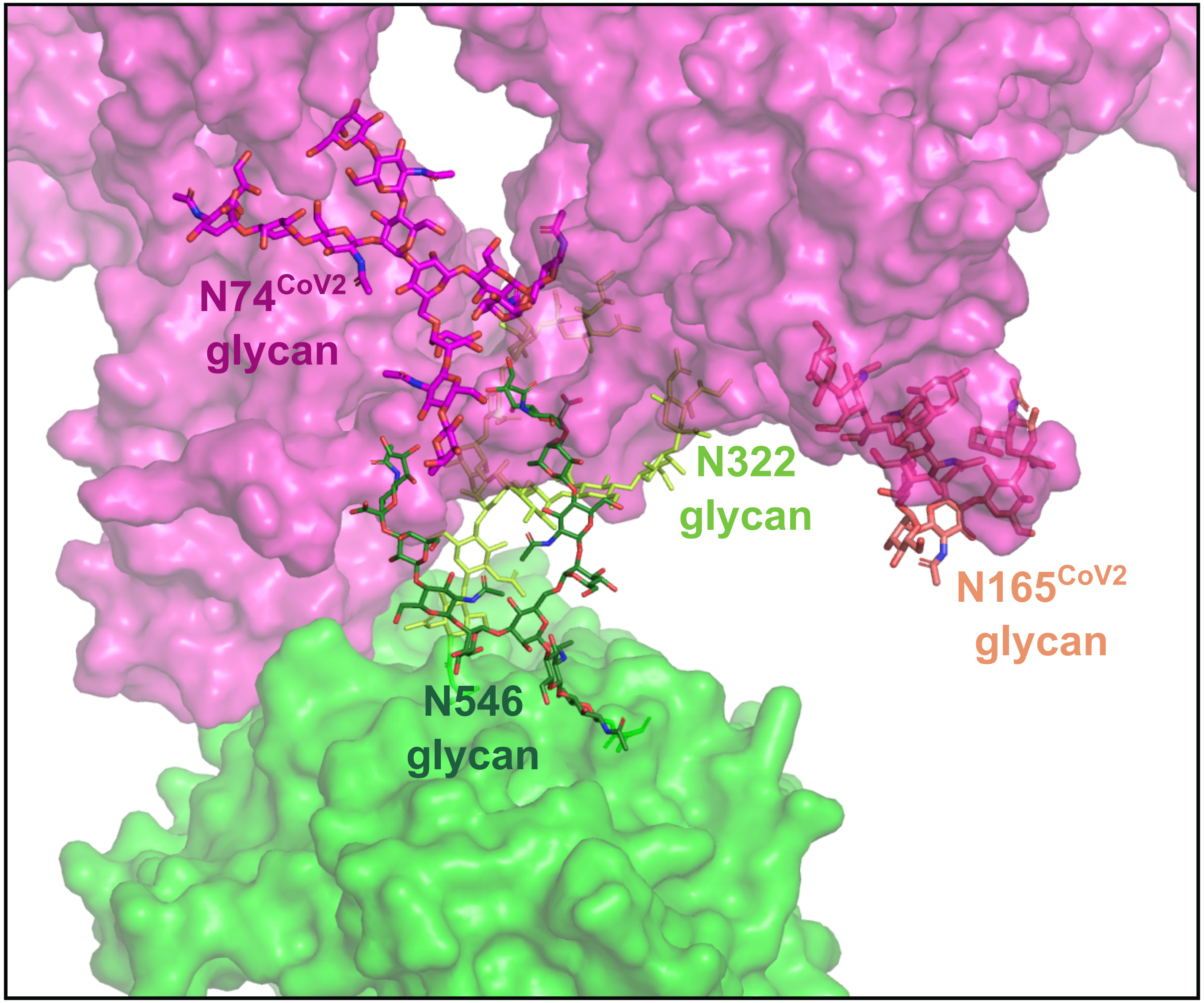

Figure S15. Interaction between the ACE2 and spike glycans. ACE2 and spike are marked by green and purple, respectively.

Table S1. Description of the different simulations performed in this study.

System name	Protein components			System size	Glycosylation	Length (ns)
	ACE	B^0AT1	RBD			
HMC_WBAT_WRBD	+	+	+	210×210×255	Homogeneous complex N-glycan	1×1000<br>2×480
HMC_WBAT_WoRBD	+	+	-	210×210×240	Homogeneous complex N-glycan	1×1000<br>2×480
HMC_WoBAT_WRBD	+	-	+	210×210×255	Homogeneous complex N-glycan	1×1000<br>2×480
HMHM_WoBAT_WRBD	+	-	+	210×210×255	Homogeneous high mannose N-glycan	1×1000<br>2×480
HMHM_WoBAT_WoRBD	+	-	-	210×210×240	Homogeneous high mannose N-glycan	1×1000<br>2×480
HT1_WoBAT_WRBD	+	-	+	210×210×255	Heterogeneous N-glycan 1	1×1000<br>2×480
HT1_WoBAT_WoRBD	+	-	-	210×210×240	Heterogeneous N-glycan 1	1×1000<br>2×480
HT2_WoBAT_WRBD	+	-	+	210×210×255	Heterogeneous N-glycan 2	1×1000<br>2×480
HT2_WoBAT_WoRBD	+	-	-	210×210×240	Heterogeneous N-glycan 2	1×1000<br>2×480
HTHS_WoBAT_WRBD	+	-	+	210×210×255	Heterogeneous N-glycan high sialic acid	1×1000<br>2×480
HTHS_WoBAT_WoRBD	+	-	-	210×210×240	Heterogeneous N-glycan high sialic acid	1×1000<br>2×480
HMC_RBD	-	-	+	125×125×125	Homogeneous complex N-glycan	1×1000

Table S2. Number and type of interactions between the N322 glycan with the putative binding site in the RBD.

Glycosylation pattern	Hydrogen bonds	Weak hydrogen bonds	Hydrophobic interactions
HMC	3.4 (2.3[a])	4.2 (4.3)	3.0 (3.5)
HMHM	3.4 (2.0)	8.5 (5.0)	3.4 (2.7)
HT1	3.1 (2.2)	6.3 (5.4)	3.3 (3.1)
HT2	3.0 (2.0)	7.4 (4.9)	3.9 (3.7)
HTHS	2.4 (2.9)	4.5 (3.2)	2.5 (3.1)

[a] standard deviation.

Table S3. The lipid composition used in this study for the membrane bilayer

	CHOL	POPC	POPE	POPS	PSM
Upper leaflet	33	33	-	-	33
Lower leaflet	20	35	25	20	-

.
